# Acute and Chronic Presentation of Melioidosis: ^18^ F-FDG PET/CT Case Series

**DOI:** 10.1055/s-0045-1809422

**Published:** 2025-06-03

**Authors:** Harini Koorma, Mohan Roop Jayanthi, Haripriya Reddy Challa, Nagamani Chella, Suneetha Batchu

**Affiliations:** 1 Department of Nuclear Medicine and PET-CT, AIG Hospitals, Hyderabad, Telangana, India; 2 Department of Infectious Diseases, AIG Hospitals, Hyderabad, Telangana, India; 3 Department of Microbiology, AIG Hospitals, Hyderabad, Telangana, India

**Keywords:** melioidosis, *Burkholderia pseudomallei*, 18-fluorine-fluorodeoxyglucose positron emission tomography–computed tomography, culture and sensitivity, pyrexia of unknown origin

## Abstract

Melioidosis is an infectious disease that is caused by a gram-negative bacillus, *Burkholderia pseudomallei* , which has been designated as a category B bioterrorism agent by the U.S. Centers for Disease Control and Prevention. Melioidosis manifests as a multi-system disorder. The effectiveness of ^18^ F-FDG PET/CT (18-fluorine-fluorodeoxyglucose positron emission tomography–computed tomography) in diagnosing or managing melioidosis is currently uncertain or not well-established. ^18^ F-FDG PET/CT is a useful tool in detecting the location and extent of abscess formation, assessing the organs involved, and detecting occult foci of infection, due to the multifocal nature of the disease. Herein, we present cases of two patients referred for a whole-body ^18^ F-FDG PET/CT scan with a history of pyrexia of unknown origin.

## Introduction

Melioidosis is an infectious disease that is caused by a gram-negative bacillus, *Burkholderia pseudomallei* , which is highly endemic in South and Southeast Asia, as well as in northern Australia, due to its widespread distribution across tropical regions. Infection is caused by inoculation, inhalation, and ingestion, 1 leading to bacteremia, which is followed by the formation of abscesses in the lungs, liver, and spleen. Because of its multi-systemic involvement and range of clinical symptoms, from acute pneumonia to chronic relapsing infection, this potentially lethal disease resembles multiple other diseases, hence known as the “great mimicker.” 2 3 Melioidosis can mimic malignancy or tuberculosis in subacute and chronic infections.

## Case 1

A 42-year-old male came with complaints of high spiking fever, jaundice, vomiting, and left joint pain for 1 month. The patient has no co-morbidities. Upon examination, the patient's condition was characterized by fever, icterus, normal blood pressure, and heightened heart and respiratory rates. On local examination, there was left knee swelling with local rise of temperature. On systemic examination, normal vesicular breath sounds were auscultated. Abdominal examination revealed no abnormalities. No cardiovascular or neurological abnormalities were detected. Routine laboratory investigations revealed deranged liver function test and mild thrombocytopenia. Blood culture and other fever workup were sent. As the patient complained of severe left knee pain, ultrasound of the knee was done, which showed mild effusion with cellulitis. The blood culture and sensitivity investigation demonstrated the isolation and growth of *B. pseudomallei* and diagnosed as melioidosis, and antibiotics are started according to the culture and sensitivity report. Whole-body 18-fluorine-fluorodeoxyglucose positron emission tomography–computed tomography ( ^18^ F-FDG PET/CT) scan was performed to search for additional areas of potential involvement and presence of internal abscesses.

**Fig. 1:**
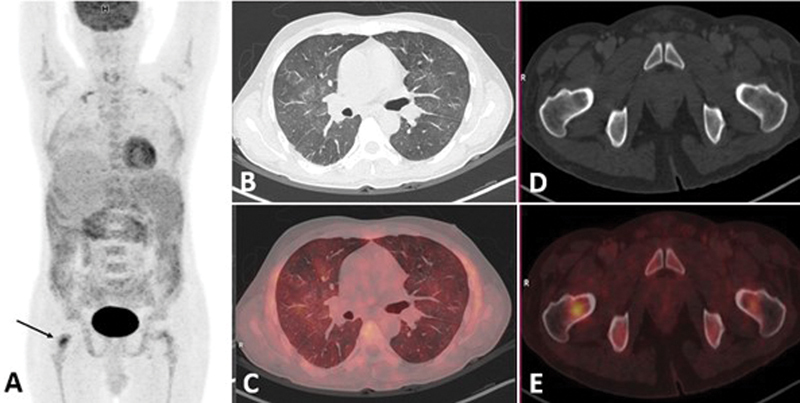
( **A** ) MIP shows focal increased ^18^ F-FDG uptake in intertrochanteric region of right femur (represented by black arrow). ( **B, C** ) Axial sections of thorax (CT; fused PET/CT) show diffuse mosaic ground glassing with subsegmental consolidation in bilateral lung fields. ( **D, E** ) Axial sections of pelvis (CT; fused PET/CT) show increased FDG uptake in intertrochanteric region of right femur. CT, computed tomography; ^18^ F-FDG, 18-fluorine-fluorodeoxyglucose; MIP, maximum intensity projection; PET, positron emission tomography.

**Fig. 2:**
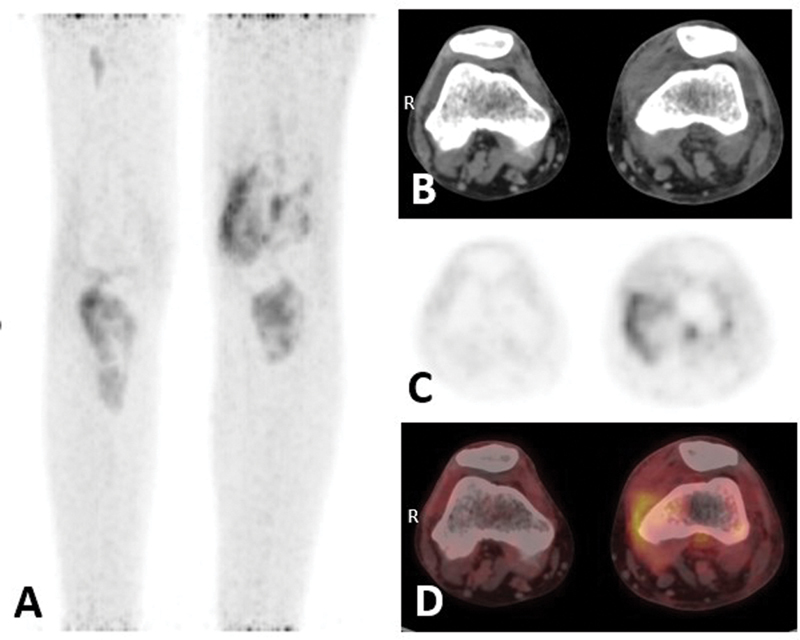
( **A** ) MIP shows increased ^18^ F-FDG uptake in left lower femoral condyles and bilateral tibia. ( **B–D** ) Axial sections of bilateral knee joint region (CT; PET; fused PET/CT) show joint effusion in the left knee with periarticular fat stranding. CT, computed tomography; ^18^ F-FDG, 18-fluorine-fluorodeoxyglucose; MIP, maximum intensity projection; PET, positron emission tomography.

**Fig. 3:**
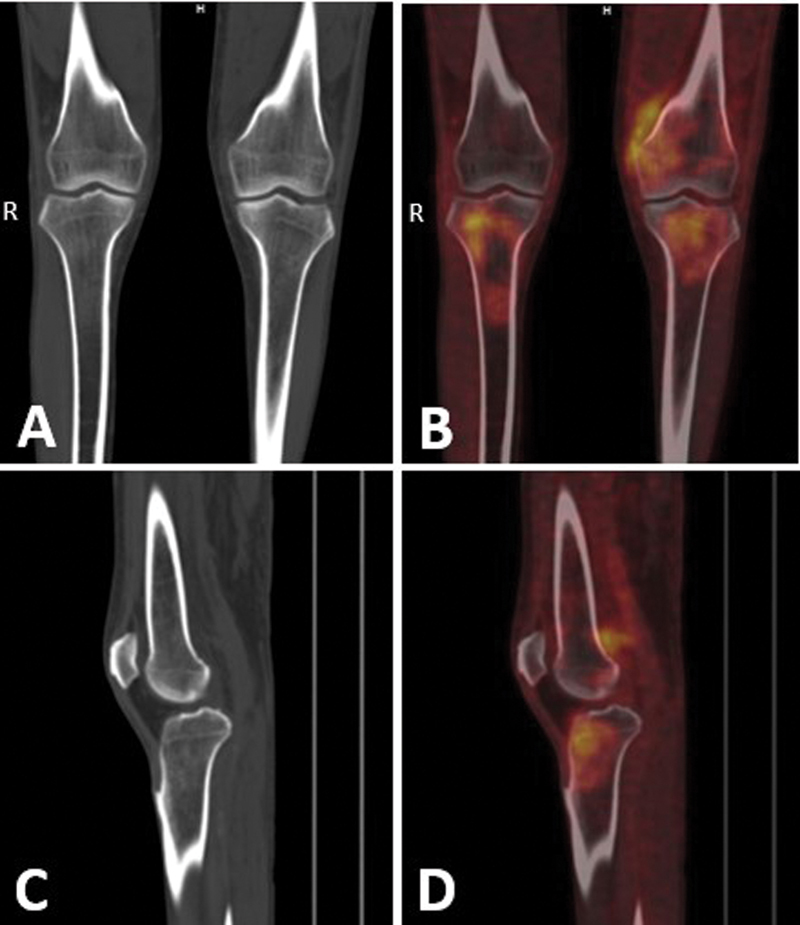
( **A–D** ) Coronal and sagittal sections of bilateral knee joint region in bone window (CT; PET; fused PET/CT) show increased metabolic activity in epiphysis and metaphysis of bilateral tibia and left lower femoral condyles, extending into adjacent diaphyseal region in right tibia with corresponding CT showing no abnormality. CT, computed tomography; PET, positron emission tomography.

^18^ F-FDG PET/CT scan revealed bilateral lung ground glass opacities with subsegmental consolidation ( Fig. 1 ) and increased FDG uptake noted in left femoral condyles with joint effusion ( Figs. 2 and 3 ). No obvious evidence of joint erosion/destruction was observed.

Throughout the hospital stay, the patient received ultrasound physical therapy for the left knee and continued with the same course of treatment after the PET/CT scan revealed no additional potential sites of solid organ involvement. The patient's symptoms improved and got discharged from the hospital.

## Case 2

A 54-year-old male presented with complaints of intermittent fever for 1.5 years which was associated with loss of appetite, weight loss, and dry cough. He is a known diabetic on regular medication for the past 10 years. The patient underwent high-resolution CT chest for intermittent fever and dry cough. The scan findings revealed consolidatory changes along with pleural effusion. Despite treatment for these findings, the patient's symptoms did not improve, leading to his referral to a higher center for evaluation and management.

**Fig. 4:**
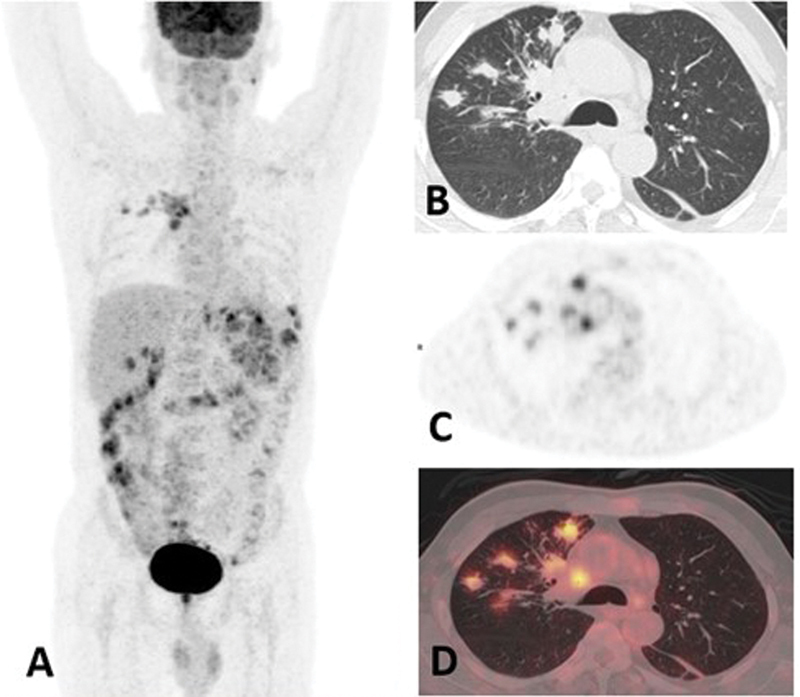
( **A** ) Maximum intensity projection (MIP) shows patchy increased 18-fluorine-fluorodeoxyglucose ( ^18^ F-FDG) uptake in the right lung, mediastinal lymph nodes, and spleen. ( **B–D** ) Axial sections of thorax (computed tomography [CT]; positron emission tomography [PET]; fused positron emission tomography–computed tomography images [PET/CT]) show patchy consolidatory changes in the right upper lobe.

**Fig. 5:**
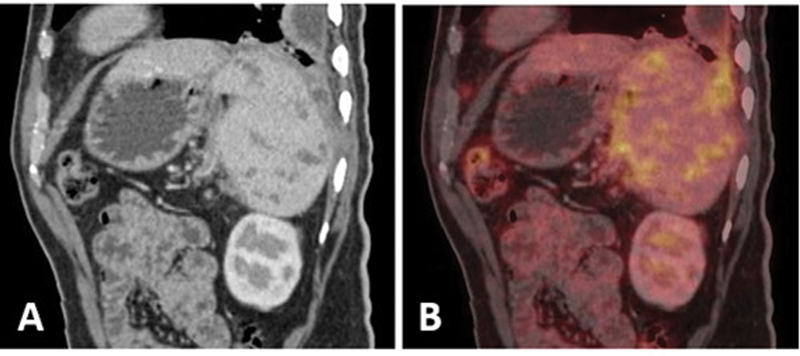
( **A, B** ) Sagittal images of abdomen (CT; fused PET/CT) show multiple ill-defined hypodense lesions in spleen showing increased ^18^ F-FDG uptake. CT, computed tomography; ^18^ F-FDG, 18-fluorine-fluorodeoxyglucose; PET, positron emission tomography.

**Fig. 6:**
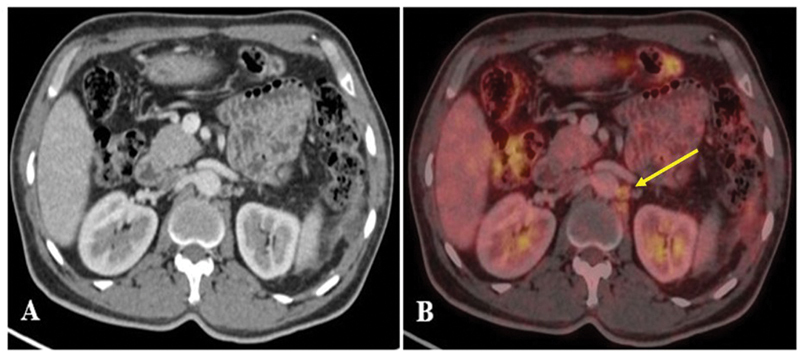
( **A, B** ) Axial section shows (CT and fused PET/CT) mildly increased ^18^ F-FDG uptake in necrotic left paraaortic lymph node (represented by yellow arrow). CT, computed tomography; ^18^ F-FDG, 18-fluorine-fluorodeoxyglucose; PET, positron emission tomography.

Laboratory tests revealed elevated erythrocyte sedimentation rate, and the count of white blood cells, specifically neutrophils, is higher than normal. The ^18^ F-FDG PET/CT scan was indicated in this case to investigate the underlying cause of pyrexia of unknown origin (PUO). The patient had been experiencing intermittent fever for 1.5 years, along with symptoms like weight loss, loss of appetite, and dry cough. Despite treatment, his condition did not improve, prompting further investigation to identify the source of the fever. PUO is a diagnostic challenge where the cause of the fever remains undiagnosed after initial investigations, and ^18^ F-FDG PET/CT is a useful imaging tool in such cases. ^18^ F-FDG PET/CT was indicated to evaluate potential infectious, inflammatory, or neoplastic causes. Whole-body ^18^ F-FDG PET/CT scan revealed metabolically active patchy consolidative changes with air bronchogram in right upper lobe, nodular left pleural thickening, mild left pleural effusion, mediastinal and abdominal lymph nodes, and ill-defined hypodense lesions noted in the spleen, few of them are conglomerate and showing internal septations ( Figs. 4 , 5 , 6 ).

**Fig. 7:**
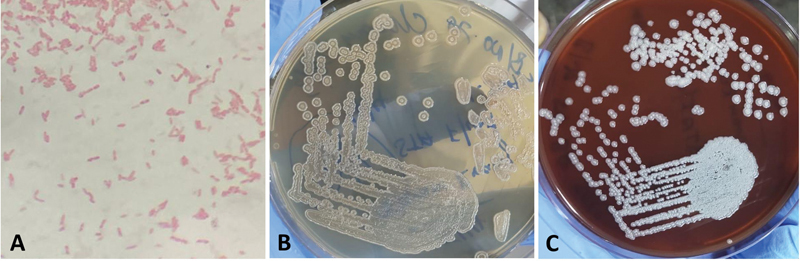
( **A–C** ) *Burkholderia pseudomallei* is a gram-negative bacillus with a characteristic bipolar or “safety pin” appearance on Gram staining. On nutrient agar and blood agar, the colonies exhibit a distinctive rough and corrugated morphology.

Based on the available information from PET/CT, CT-guided biopsy from the metabolically active splenic lesions was performed. *Burkholderia pseudomallei* was isolated in the culture specimen. These organisms are gram-negative bacilli that typically exhibit bipolar or safety pin appearance. *Burkholderia pseudomallei* is an obligate aerobe which grows on blood agar, nutrient agar, and MacConkey agar. These colonies are typically rough and corrugated ( Fig. 7 ).

## Discussion

Gram-negative *B. pseudomallei* is a naturally occurring organism found in soil and surface groundwater. 4

The most prevalent risk factor is diabetes mellitus; other recognized risk factors include male sex; age >45 years; alcoholism; chronic lung, kidney, and liver diseases; hematological disorders such as thalassemia; immunosuppression; and prolonged use of steroids.

Melioidosis can manifest clinically as acute (∼85%), subacute, or chronic (over 2 months, typically less than 12%) processes. 5 In our scenario, case 1 exhibited an acute presentation while case 2 displayed a chronic presentation.

Patients with pneumonia (51%), genitourinary infections (14%), skin infections (13%), bacteremia without obvious focus (11%), septic arthritis or osteomyelitis (4%), and neurologic involvement (3%) were the main presenting features in a descriptive study that involved 500 patients over the course of 20 years in tropical Australia. A clear focus of infection was absent in the remaining 4% of patients. 6

Gopalakrishnan et al 7 discovered 32 cases of melioidosis with confirmed cultures. According to their findings, the majority of cases of infection presented with fever and weight loss, and the incidence was higher in males with a median age of 42.5 years.

In endemic locations, melioidosis has a high fatality rate of 19 to 36%, 8 with worldwide mortality rates ranging from 9 to 70%. 9

The most frequently impacted organ by melioidosis is the lung. Initially it starts with multiple small pulmonary nodules and multilobar infiltrates in the upper lobes and they progress to mixed nodular and patchy alveolar opacities in subacute and chronic forms. 10

The liver, kidney, and spleen are the next most frequently affected visceral organs in the abdomen. The presence of hepatic and splenic abscess together is highly suggestive of melioidosis. 11

Musculoskeletal, prostatic involvement, and neurological manifestations are the other manifestations seen in melioidosis. 12 13 14

Melioidosis mimics a variety of clinical conditions in both radiological and clinical aspects. It is often misdiagnosed in India as tuberculosis due to the striking similarities in clinical presentations. In these cases, pulmonary tuberculosis should be ruled out as a differential diagnosis. The two most significant characteristics that could differentiate melioidosis from tuberculosis are frequent presentations with concurrent liver and splenic abscesses and sparing of the lung apex. Fascinatingly, pleural involvement—such as effusion, empyema, hilar or mediastinal adenopathy—is less common in melioidosis, which makes it possible to distinguish it from tuberculosis with additional useful characteristics. 15 In case 2, however, there were nodular left pleural thickening, mild left pleural effusion, and mediastinal lymph nodes—all of which are comparatively less common in melioidosis and make it challenging to differentiate it from tuberculosis. The gold standard for *B. pseudomallei* diagnosis is culture and sensitivity.

^18^ F-FDG PET/CT is a sensitive, noninvasive hybrid imaging technique used in suspected cases of infection or inflammation. 16 To get histopathological results and a final diagnosis, ^18^ F-FDG PET/CT is helpful to identify possible fever of unknown lesions and also to guide the site of biopsy. In case 2 scenario, ^18^ F-FDG PET/CT helped in locating the possible site for biopsy. ^18^ F-FDG PET/CT is also used for response evaluation to antibiotic therapy. There are several studies which had reported the use of ^18^ F-FDG PET/CT for the management of *B. pseudomallei* infection. 17 18 19

Melioidosis cannot be diagnosed based only on clinical characteristics. Overall, the most reliable method for diagnosing melioidosis is the culture-based approach which is considered conclusive.

## Conclusion

^18^ F-FDG PET/CT is a valuable tool in diagnosing and managing melioidosis, aiding in the detection of metabolically active lesions and identifying the extent of organ involvement. In these cases, PET/CT revealed critical findings, including lung, pleural, and joint involvement, which guided further diagnostic workup and treatment. The scan's ability to identify systemic infection and guide biopsy for microbiological confirmation was pivotal in diagnosing *B. pseudomallei* . Given its ability to assess both the metabolic activity and spread of infection, FDG PET/CT should be considered an essential diagnostic adjunct in complex or refractory melioidosis cases.

## Notes

**Corrigendum:** This article has been corrected as per the corrigendum published online on June 26, 2025 (doi: 10.1055/s-0045-1809982).
